# Effects of Early Treatment with Lipid Core Nanoparticles-Associated Methotrexate on Cardiac Remodeling and Soleus Muscle Inflammasomes in Infarcted Rats

**DOI:** 10.3390/ijms27094140

**Published:** 2026-05-06

**Authors:** Anna Clara C. Santos, Mariana Gatto, Gustavo A. F. Mota, Patrícia A. Borim, Rafael C. F. Silva, Ana Luisa B. Meirelles, Lidiane M. Souza, Elida P. B. Ojopi, Eder A. Rodrigues, Luana U. Pagan, Ana Paula S. Marreiros, Gabriela Brandao, Leonardo A. M. Zornoff, Raul C. Maranhão, Katashi Okoshi, Marina P. Okoshi

**Affiliations:** 1 Internal Medicine Department, Botucatu Medical School, Sao Paulo State University (UNESP), Botucatu 18618-687, SP, Brazil; anna.consorti@unesp.br (A.C.C.S.); mariana.gatto@unesp.br (M.G.); gustavo.mota@unesp.br (G.A.F.M.); patricia.borim@unesp.br (P.A.B.); rafael.silva@unesp.br (R.C.F.S.); ana.meirelles@unesp.br (A.L.B.M.); lidiane.souza@unesp.br (L.M.S.); eder.rodrigues@unesp.br (E.A.R.); luana.pagan@unesp.br (L.U.P.); ana.marreiros@unesp.br (A.P.S.M.); gabriela.brandao@unesp.br (G.B.); leonardo.zornoff@unesp.br (L.A.M.Z.); katashi.okoshi@unesp.br (K.O.); 2 Clinic Hospital, Botucatu Medical School, Sao Paulo State University (UNESP), Botucatu 18618-687, SP, Brazil; benquique.ojopi@unesp.br; 3 Faculty of Pharmaceutical Sciences, University of Sao Paulo, Sao Paulo 05508-000, SP, Brazil; ramarans@usp.br

**Keywords:** myocardial infarction, inflammation, NLRP1, NLRP3, NLRC4, skeletal muscle, ventricular function, anti-inflammatory drug, heart failure

## Abstract

Substances released by cardiomyocytes after myocardial infarction (MI) lead to inflammasome assembly. Heart failure (HF) is associated with skeletal muscle inflammation. Methotrexate (MTX) reduces cardiovascular outcomes in chronic inflammation patients. Lipid core nanoparticle-associated MTX (MTX-LDE) attenuated cardiac remodeling in MI rats. We investigated the effects of early MTX-LDE administration on cardiac remodeling and inflammasomes in soleus muscle of MI rats. Wistar rats were separated into Sham, MI, and MI-MTX groups. MTX was initiated 24 h after MI at 1 mg/kg/week intraperitoneally for 10 weeks. Soleus protein expression of NLRP1, NLRP3, NLRC4, ASC, procaspase-1, Caspase-1, pro-IL-1β, and IL-1β was quantified by Western blotting; Nlrp1a, Nlrp3, Nlrc4, Pycard (Asc), Casp1, and Il1b gene expression was assessed by qPCR; and statistical analysis used Student’s t test and ANOVA. Rats with infarction size > 35% total left ventricle (LV) area were included in the study; infarction size did not differ between groups. Echocardiogram showed infarcted groups with LV dilation and dysfunction. Diastolic function was worse in MI-MTX than MI. NLRP1 and NLRC4 protein expression was lower in MI-MTX than Sham. Expression of other proteins and gene expression did not differ between groups. Early MTX-LDE administration reduces NLRP1 and NLRC4 protein expression in soleus muscle without improving cardiac remodeling in rats.

## 1. Introduction

Myocardial infarction (MI) is a common cause of death in cardiovascular disease [1]. Patients who survive acute MI are at increased risk of developing cardiac remodeling and heart failure (HF) [2]. Dying cardiomyocytes release danger-associated molecular patterns (DAMPs) after myocardial ischemia; these are recognized by nucleotide-binding receptors (NLRs) in the myocardium. This recognition triggers myocardial inflammation characterized by cytokine activation and necrotic cell clearance [3,4]. Although inflammation is essential for injured cardiac tissue cicatrization, excessive and prolonged inflammation can aggravate cardiac remodeling and worsen outcomes [5].

NLRs can assemble into a multimolecular complex known as the inflammasome, which acts to amplify the acute inflammatory response. The most extensively studied inflammasomes include NLRP3, which means NLR (nucleotide-binding domain and leucine-rich repeat containing protein) family pyrin domain-containing protein 3; NLRP1 (NLR family pyrin domain-containing protein 1); and NLRC4 (NLR family caspase recruitment domain-containing protein 4) [4,6]. Inflammasomes are formed by an NLR, an apoptosis-associated speck-like protein containing a caspase recruitment domain (ASC) and Caspase-1 (CASP1), which cleaves and activates the pro-inflammatory cytokine interleukin (IL)-1β [7,8]. As the most widely investigated, NLRP3 expression has been well documented in cardiac fibroblasts [4,9,10]. The fact that myocardium exhibits increased NLRP3 inflammasome levels post-acute MI supports the role of NLRP3 in mediating myocardial cell death after MI [11]. In contrast, the roles of NLRP1 and NLRC4 in post-MI inflammation remain unclear [12].

Heart failure induces significant alterations in skeletal muscle, known as heart failure-induced skeletal myopathy [13,14,15]. Clinical and experimental studies have reported that advanced heart failure is associated with skeletal muscle atrophy, myocyte apoptosis, and inflammation [16,17]. Skeletal myocyte apoptosis can activate immune response, including IL-1β upregulation, which has been implicated in skeletal muscle atrophy [9,18].

Methotrexate (MTX), an immunosuppressive drug, is widely used in the treatment of cancer and autoimmune diseases such as rheumatoid arthritis [19,20]. MTX has been shown to reduce cardiovascular outcomes in chronic inflammation patients and inhibit NLRP3 activation [19,21,22,23]. Lipid core nanoparticle-carried MTX (MTX-LDE) enhanced drug efficacy and bioavailability and reduced cardiac remodeling in infarcted rats [24,25,26].

Although some studies have explored the involvement of inflammasomes in skeletal muscle diseases [9,27], their role following acute MI and the effect of MTX-LDE on skeletal muscle inflammasomes have not been established. This study aimed to evaluate NLRP1, NLRP3, and NLRC4 expression in skeletal muscle following early MTX-LDE treatment after acute MI in rats. We also assessed the expression of other inflammasome components including caspase-1, ASC (apoptosis-associated speck-like protein containing a CARD; encoded by Pycard), and IL-1β.

## 2. Results

**Figure 1:**
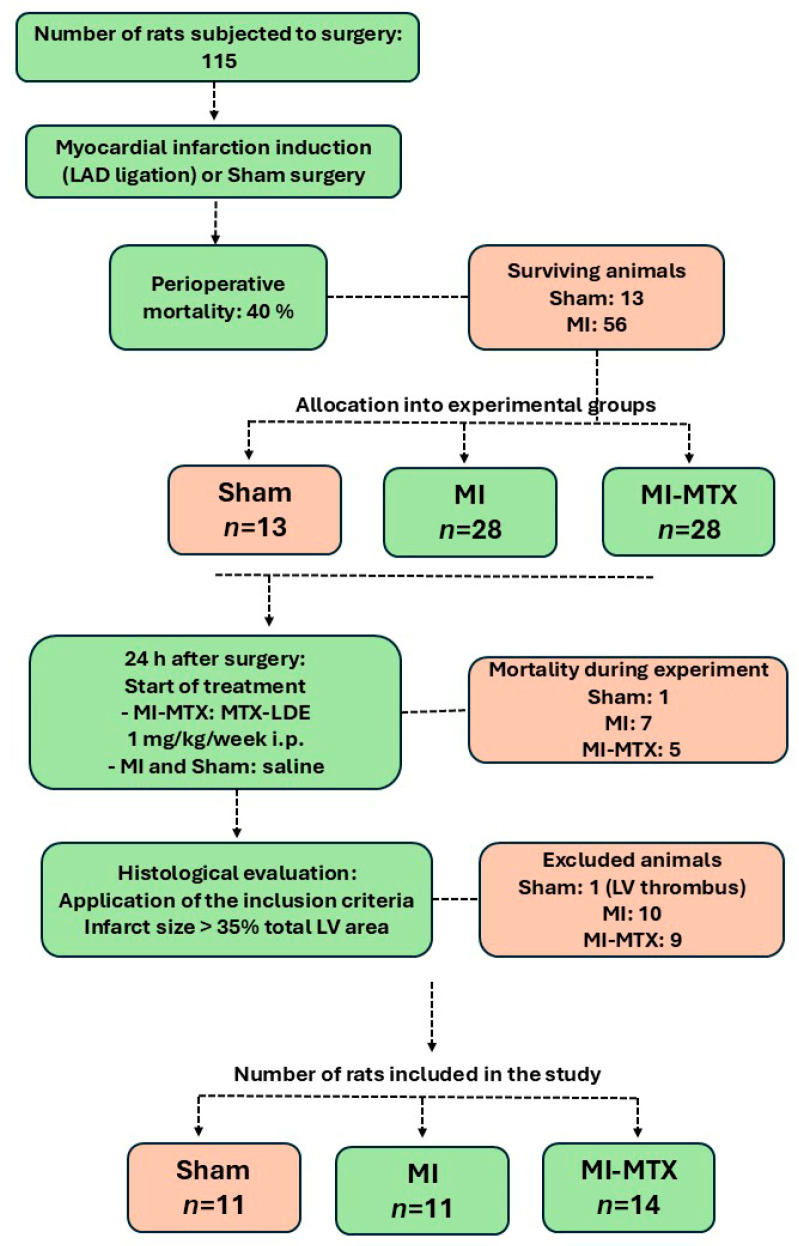
Flow diagram of the experimental protocol. Representation of animal groups, treatment, mortality, and exclusion criteria. MI: myocardial infarction; MI-MTX: MI treated with methotrexate; LAD: left anterior descending coronary artery; MTX-LDE: lipid core nanoparticle-carried MTX; i.p.: intraperitoneal; LV: left ventricle.

A flowchart of the study is shown in Figure 1. Peri-operative mortality rate was 40%. Treatment was initiated 24 h after surgery. Infarct size was evaluated post-mortem by histology; to ensure a significant degree of cardiac injury, only rats with infarct size greater than 35% total left ventricle (LV) area were included in the study.

### 2.1. Cardiac Structural and LV Function Evaluated by Echocardiogram

At the end of the experiment, infarct size, also assessed by echocardiogram, did not differ between MI-MTX and MI groups. MI-MTX and MI had left ventricle (LV) and left atrium dilation compared to Sham (Table 1). Echocardiographic structural parameters did not differ between MI-MTX and MI. MI-MTX and MI had systolic dysfunction, characterized by lower endocardial fractional shortening, posterior wall shortening velocity, ejection fraction, area variation, and tissue Doppler imaging of mitral annulus systolic velocity (TDI S’); and diastolic dysfunction, characterized by higher mitral E wave and E/A ratio than Sham. Diastolic function was worse in MI-MTX than MI, characterized by higher mitral E wave and E/A ratio than MI, and lower E-wave deceleration time than Sham. Tei index, a useful parameter for assessing both systolic and diastolic dysfunction, was higher in MI-MTX and MI than Sham (Table 2).

### 2.2. Anatomical Parameters

LV weight was lower in MI-MTX than MI, while RV weight was higher in MI-MTX than Sham and MI groups. RV/BW ratio was higher in MI-MTX and MI than Sham, and higher in MI-MTX than MI. Atria weight was higher in both infarcted groups than Sham. Right and left soleus muscle weights did not differ between groups (Table 3).

### 2.3. Histological Analysis

**Figure 2:**
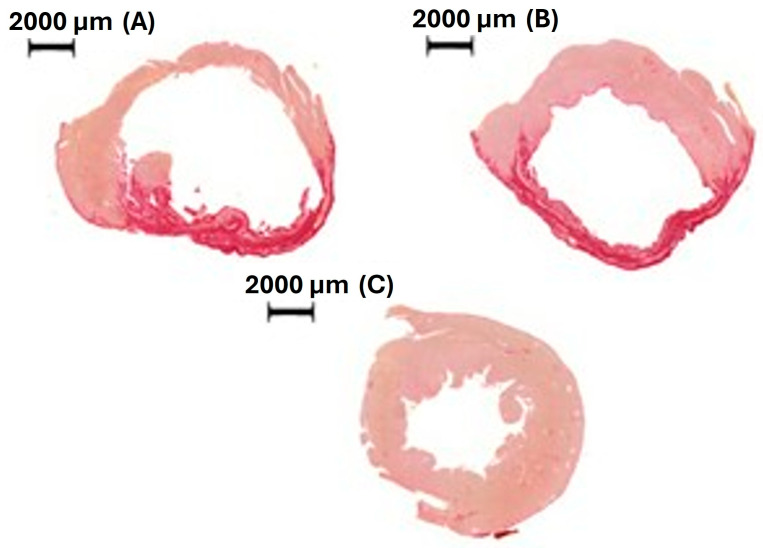
Representative picro-sirius red-stained histological sections showing left ventricle from myocardial infarction; MI (**A**), MI treated with methotrexate (**B**), and Sham (**C**) groups. The histochemical picro-sirius red staining is used to identify collagen fibers (fibrosis) in myocardial tissue. Under brightfield microscopy, it stains collagen red, while muscle fibers appear pale yellow or pale rose. The large infarcted area is shown in both MI (**A**) and MI treated with methotrexate (**B**) groups.

**Figure 3:**
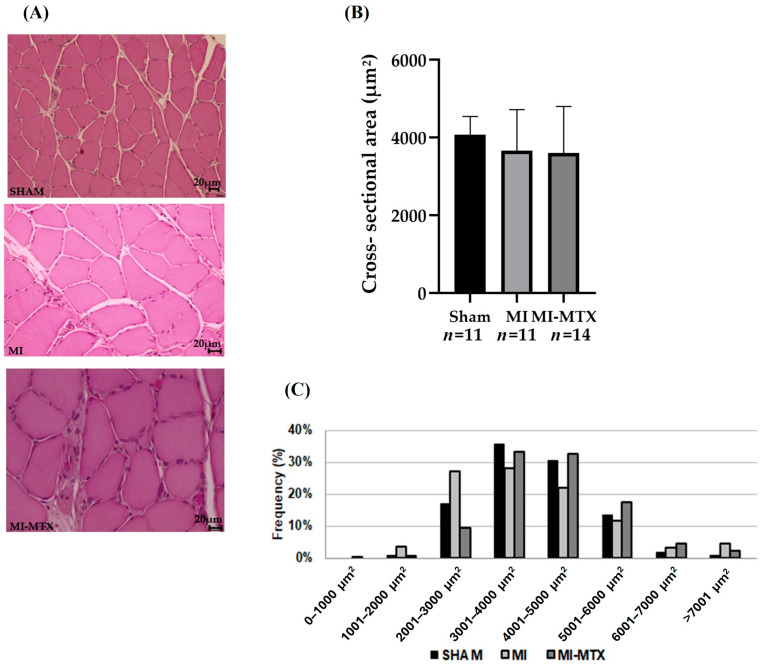
Soleus muscle morphometry. Representative hematoxylin–eosin-stained histological sections (**A**); cross-sectional area, means ± standard deviation (**B**); and frequency representing distribution of fibers according to size (**C**). MI: myocardial infarction; MI-MTX: MI treated with methotrexate; *n*: sample size. ANOVA and Bonferroni; *p* > 0.05.

Infarct size did not differ between groups (MI 50 ± 7.1%; MI-MTX: 50 ± 10.5% of total LV area; *p* > 0.05; Figure 2). Soleus muscle fiber myocyte cross-sectional area did not differ between groups. Fiber cross-sectional area distributions are shown in Figure 3.

### 2.4. Protein Expression

**Figure 4:**
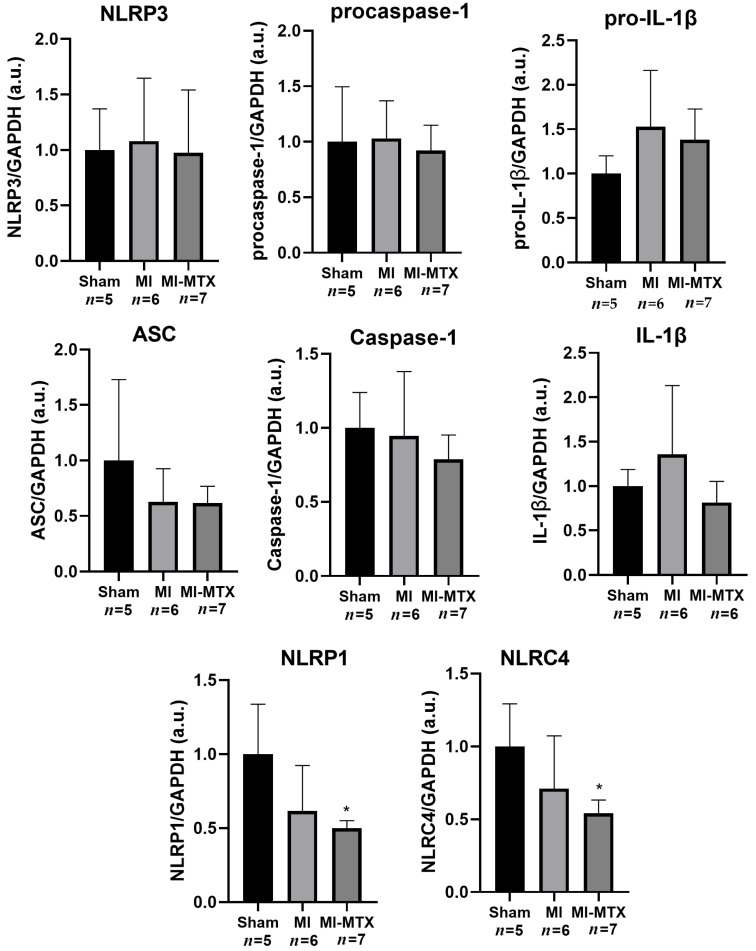
Protein expression of inflammasome components in soleus muscle analyzed by Western blot. Data are expressed as means ± standard deviation. NLRP3: NACHT, LRR, and PYD domain-containing protein 3; pro-IL-1β: pro-interleukin-1 beta; ASC: apoptosis-associated speck-like protein containing a caspase recruitment domain; IL-1β: interleukine-1 beta; NLRP1: NACHT, LRR, and PYD domain-containing protein 1; NLRC4: NLR family CARD domain-containing protein 4; MI: myocardial infarction; MI-MTX: MI treated with methotrexate; *n*: sample size. ANOVA and Bonferroni; * *p* < 0.05 vs. Sham.

Protein expressions of NLRP3, ASC, Procaspase-1, Caspase-1, pro-IL-1β, and IL-1β did not differ between groups. NLRP1 and NLRC4 protein expression was lower in MI-MTX than Sham (Figure 4). Detailed blot images are presented in the Supplementary Materials Figure S1.

### 2.5. Gene Expression

mRNA expressions of Nlrp3, Nlrp1a, Nlrc4, Pycard, Casp1, and Il-1β did not differ between groups.

## 3. Discussion

This study evaluated the effect of early administration of methotrexate carried in lipid core nanoparticles on cardiac remodeling and skeletal muscle inflammasome modulation 10 weeks after myocardial infarction in rats.

MI in rats is a well-established model for evaluating the pathophysiology and treatment of cardiac remodeling due to practicality, low costs, and good reproducibility of results. As we have previously observed that the minimum LV infarct size to induce chronic ventricular dysfunction is approximately 38% [28], only rats with an infarcted area >35% total LV area were included in the study. This criterion ensured an established cardiac injury model. Infarct size homogeneity between infarcted groups, confirmed by echocardiogram and histological analyses, further reinforced the reliability of the experimental model.

Methotrexate, considered the gold standard for treating rheumatoid arthritis, has shown potential for improving cardiovascular outcomes in patients with chronic inflammation [19,29]. A retrospective observational study reported a reduced risk for developing heart failure in rheumatoid arthritis patients treated with MTX [30]. However, in patients with stable atherosclerosis without rheumatologic disease, low-dose methotrexate did not reduce levels of inflammatory markers or cardiovascular events compared to placebo [31]. Concerning cardiac remodeling, studies have failed to show beneficial effects of MTX on postischemic cardiac damage in both humans and rodents after acute MI [32,33]. Therefore, the role of MTX on cardiovascular outcomes and cardiac remodeling is not completely established.

To enhance therapeutic efficacy, MTX was recently associated with lipid core nanoparticles which are recognized by low-density lipoprotein (LDL) receptors in injured cells. This approach allows targeted delivery with increased drug concentration at the injury site [24,25]. The formulation was tested in vitro, in vivo, and in small clinical trials. Increased uptake of MTX-LDE was observed in cultured neoplastic cells and in malignant tissue of cancer patients [34,35,36,37,38]. Also, myocardial uptake of MTX-LDE was higher in non-infarcted areas from infarcted rats than in Sham [26], supporting the rationale for its use under systemic inflammation.

Early post-MI periods are characterized by an inflammatory process that is essential for tissue repair but may become detrimental if excessive or prolonged [5]. Initiating treatment 24 h post-MI was aimed at targeting the peak of the early inflammatory phase to modulate pro-inflammatory signaling and prevent the progression to pathological fibrosis. Previously, MTX-LDE administration 24 h post-acute MI was shown to improve cardiac remodeling and systolic function in rats [26]. However, our study, which also initiated MTX-LDE 24 h post-acute MI, MTX-LDE did not show improved cardiac remodeling or LV function. In fact, when observing all data relative to post-MI cardiac remodeling, we could observe a trend for MTX-LDE to impair cardiac remodeling as MI-MTX had higher E/A ratio and right ventricle weight than MI, parameters that are often used to detect diastolic dysfunction in rats. Thus, additional studies are needed to clarify these discrepant results.

Chronic heart failure is often associated with muscle mass loss, which is characterized by reduced protein synthesis and increased protein degradation [17,39]. Danger-associated molecular patterns (DAMPs) have been implicated in triggering muscle atrophy during heart failure [40]. We did not observe soleus muscle atrophy in both muscle weight and cross-sectional area. This suggests that, despite the presence of systolic and diastolic dysfunction in both infarcted groups, the rats have not progressed to severe clinical heart failure, and thus skeletal muscle changes were not fully manifested.

Inflammasomes play a central role in amplifying inflammatory response following MI [4,41]. NLRP3 expression is established in cardiomyocytes post-MI; furthermore, its inhibition reduced infarct size and improved cardiac function in rodents [42]. However, the involvement of inflammasomes in heart failure-induced skeletal muscle changes remains unclear even though necrosis, inflammation, and atrophy in post-MI rats with overt heart failure have been well-documented in rat soleus muscle [17]. We have not identified any studies evaluating inflammasome expression in skeletal muscles post-MI. We observed no differences in Nlrp3 protein or gene expression in soleus muscle. You et al. [43] showed that NLRP3 inflammasome is activated and stimulates muscle atrophy during denervation, while genetic knockout of muscle NLRP3 attenuates atrophy.

ASC which recruits procaspase-1 [4] and Caspase-1, responsible for cleaving IL-1β [7,8,44], are both essential for inflammasome assembly. Increased ASC methylation has been associated with reduced plasma IL-1β levels, suggesting a potential pathway for modulating inflammation [41]. However, ASC and Caspase-1 expressions did not change in our study. Caspase-1 activity peaks at seven days post-MI and declines fourteen days later [45]. The fact that our analyses were performed ten weeks post-MI may explain the unchanged Caspase-1 expression.

IL-1β, a key proinflammatory cytokine produced upon inflammasome activation, was also evaluated [8,46]. Studies have shown that IL-1β suppression mitigates muscle mass loss and fibrosis in heart failure patients and animal models [27,47]. However, MTX-LDE did not significantly modulate skeletal muscle IL-1β expression in our study.

Although NLRP1 and NLRC4 inflammasomes are commonly associated with bacterial and viral infections [42,48], emerging evidence suggests their involvement in cardiac remodeling. Studies have shown increased myocardial NLRP1 expression in infarcted mice [49] and reduced hypertrophy, inflammation, and fibrosis in NLRP1-deficient mice with aortic stenosis [50]. Similarly, elevated myocardial NLRC4 expression has been observed in infarcted mice and heart failure patients [51,52]. However, the roles of NLRP1 and NLRC4 in skeletal muscles remain poorly understood. Our study showed for the first time that early MTX-LDE treatment reduced soleus NLRP1 and NLRC4 protein expression in MI-MTX compared to Sham. These results provide insight into the MTX-LDE anti-inflammatory potential, although the mechanistic link remains to be established. In contrast, gene expressions of these inflammasomes did not differ between groups. Protein expression is influenced not only by mRNA levels, but also by post-transcriptional and post-translational mechanisms, including translation efficiency, protein synthesis rate, and degradation pathways [53]. Therefore, our results suggest a lack of correlation between mRNA and protein levels. As previous transcriptome analyses have reported low expression of the rat skeletal muscle genes evaluated in this study, it is possible that the sensitivity of our methods was not sufficient for detecting changes at the mRNA level [54].

This study had some limitations. Firstly, as an exploratory study, the mechanistic link between MTX-LDE treatment and reduced NLRP1 and NLRC4 protein expression was not clarified. Other limitations include the lack of myocardial histological analysis and the absence of an MTX-treated Sham group.

In summary, early MTX-LDE administration did not improve post-MI cardiac remodeling evaluated by anatomical and echocardiographic parameters. The lower NLRP1 and NLRC4 protein expression in MI-MTX compared to Sham shows that MTX-LDE decreased inflammatory markers in soleus muscle. However, the muscle phenotype did not differ between groups. Muscle atrophy is the most common alteration observed in advanced heart failure [17,40]. Thus, it is probable that, despite evident echocardiographic changes, post-infarction cardiac remodeling was not long enough to induce muscle atrophy in our rats. Further studies are needed to clarify the functional relevance of our findings and the potential therapeutic implications in cardiovascular disease.

## 4. Materials and Methods

### 4.1. Experimental Design

Male Wistar rats (200–250 g) were purchased from the Central Animal Center, Botucatu Medical School, Sao Paulo State University, UNESP, Brazil. They were collectively housed, three rats per cage, in a room under controlled temperatures (24 ± 2 °C) and 12 h light–dark cycles. Food and water were provided ad libitum. Rats were weighed weekly to calculate drug dose. All experiments were approved by Botucatu Medical School (UNESP) Animal Ethics Committee (protocol number 1324/2019) and were conducted in accordance with the Guide for the Care and Use of Laboratory Animals published by the National Research Council, 2011.

A flowchart of the study is shown in Figure 1. In total, 115 rats were subjected to either MI induction surgery or a Sham operation. MI was induced by ligation of the left anterior descending coronary artery as previously described [55]. Perioperative mortality was 40%. After 24 h following surgery, surviving rats were randomly divided into three groups: Sham (*n* = 13), MI (*n* = 28), and MI-MTX (*n* = 28). The MI-MTX group received MTX-LDE 24 h after surgery at 1 mg/kg intraperitoneally once a week for 10 weeks. MTX-LDE was provided by Dr. Raul C. Maranhao (Heart Institute, INCOR, Sao Paulo University, USP). Sham and MI received 100 μL 0.9% saline solution. Dosage was based on previous studies and clinical guidelines to ensure efficacy while minimizing MTX toxicity [26,33].

At the end of the experiment, rats were subjected to transthoracic echocardiograms and euthanized the next day. Infarct size was determined by echocardiogram and histology; only rats with infarct sizes greater than 35% total LV area according to histological analysis were included in the final analysis.

### 4.2. MTX-LDE Preparation and Characterization

MTX-LDE was synthesized at the Laboratory of Metabolism and Lipids, Faculty of Pharmaceutical Sciences, University of Sao Paulo, Brazil. It is a lipid mixture comprising 100 mg of cholesteryl oleate, 200 mg of egg phosphatidylcholine (Lipoid GMBH, Ludwigshafen, RP, Germany), 10 mg of triglycerides, 12 mg of cholesterol, and 60 mg of MTX. To improve stability between LDE and MTX, MTX was esterified with dodecyl bromide, creating a lipophilic derivative. The particles have a diameter of approximately 60 nm and remain stable for 45 days at 4 °C [34,56].

### 4.3. Echocardiogram

After anesthesia by an intraperitoneal (IP) injection of ketamine (50 mg/kg) and xylazine (1 mg/kg), a transthoracic echocardiogram was performed to analyze heart structure and LV function. We used a commercial echocardiograph (General Electric Medical Systems, model Vivid S70, Tirat Carmel, Hi, Israel) equipped with a multifrequency transducer from 5.0 to 11.5 MHz, as previously described in our laboratory [57,58,59].

### 4.4. Tissue Collection

Animals were weighed and euthanized with thiopental IP (100 mL/kg) and then submitted to median thoracotomy for heart removal. Atria and ventricles were dissected and weighed separately [60]. Right and left posterior soleus muscles were then removed. All tissues intended for molecular analysis were frozen in liquid nitrogen and storage at −80 °C.

### 4.5. Histological Analysis

Left ventricle samples were cut 6 mm from the LV apex, kept in a 10% formalin solution for 24 h, washed in running water, transferred to a 70% ethanol solution, and stained with picro-sirius red. Theses slides were used to assess infarction size. The histochemical technique picro-sirius red staining is used to identify collagen fibers (fibrosis) in myocardial tissue. Under brightfield microscopy, it stains collagen red, while muscle fibers appear pale yellow or pale rose. Serial transverse soleus muscle sections were cut and stained with hematoxylin and eosin. At least 150 fiber cross-sectional areas were measured from each muscle. Fiber cross-sectional areas were separated into ranges to show the distribution of fibers according to their size. Measurements were performed using a Leica DM LS microscope (Leica Microsystems, Wetzlar, HE, Germany) attached to a computerized imaging analysis system (Media Cybernetics, Silver Spring, MD, USA).

### 4.6. Protein Expression

Protein expression was evaluated in soleus muscle by Western blotting using the primary antibodies of anti-NLRP1 (1:200, F-9, sc-166368, Santa Cruz Biotechnology Inc., Dallas, TX, USA), NLRC4 (1:500, 4B7B7, Thermo Fisher Scientific, Carlsbad, CA, USA), NLRP3 (1:200, sc06-23, Thermo Fisher Scientific), ASC (1:200, F-9, sc-271054, Santa Cruz Biotechnology), Caspase-1 (1:200, 14F468, sc-56036, Santa Cruz Biotechnology), and IL-1β (1:200, 11E5, sc52012, Santa Cruz Biotechnology) as previously described [61]. Protein concentrations were normalized to GAPDH (1:10000, 6C5, sc-32233, Santa Cruz Biotechnology). Proteins were obtained from soleus samples (~50 mg), and homogenized using RIPA with phosphatases and proteases, as well as zirconium beads (0.5 mm) for 5 min at 4 °C in a Bullet Blender^®^ homogenizer (Next Advance, Inc., Troy, NY, USA). The samples were then centrifuged for 10 min at 4 °C in 12,000 rpm. Supernatant protein was collected and quantified by BCA protein assay kit (Thermo Fisher Scientific, Waltham, MA, USA). Proteins were separated by electrophoresis in polyacrylamide gel and transferred to a nitrocellulose membrane. After 1 h blockade at room temperature with 5% non-fat dry milk in TBS-T, primary antibodies were incubated overnight at 4 °C. Membranes were then washed with PBS and Tween 20 and incubated with secondary antibodies of anti-rabbit IgG-HRP (Abcam, Cambridge, MA, USA) or anti-mouse IgG-HRP (Abcam) for 90 min at room temperature. Immobilon^®^ Classico Western HRP Substrate (Merck Millipore, Ref. WBLUC0500, Burlington, MA, USA) and an ImageQuantTM 800 (IQ800) ©Cytiva, USA, were used to detect bound antibodies, which were quantified by ImageJ V1.53t (National Institutes of Health, Bethesda, MD, USA) and are available at https://imagej.net/ij (accessed on 10 October 2023).

### 4.7. Gene Expression

Soleus gene expression of Nlrp3, Nlrc4, Nlrp1a, Casp1, Pycard (Asc) and Il-1β was analyzed by real-time quantitative reverse-transcription polymerase chain reaction (RT-PCR) as previously described [55]. Aliquots of cDNA were subjected to RT-PCR using a customized assay containing sense and antisense primers and Taqman probes (Thermo Fisher Scientific, Waltham, MA, USA) specific for each gene: Nlrp3 (Rn04244620m1), Nlrc4 (Rn1473278_m1), Nlrp1a (Rn01467482_m1), Casp1 (Rn00562724_m1), Asc (Rn00597229_g1), and Il-1β (Rn01514151_m1). Data were normalized using reference genes cyclophilin (Rn00690933_m1) and Gapdh (Rn01775763_g1). Detailed information on the assays can be accessed through the manufacturer’s database. Reactions were performed in triplicate and results calculated using the comparative CT method (2^−∆∆CT^).

### 4.8. Statistical Analysis

Parametric data are presented as means ± standard deviation and non-parametric data shown as medians and percentiles. Comparisons between groups were performed using one-way analysis of variance (ANOVA) followed by Bonferroni’s test or Kruskal–Wallis followed by Dunn’s test. Infarct size was analyzed by unpaired Student’s t test. The analyses were performed using Sigma Plot 12.0 (Systat software Inc., Palo Alto, CA, USA) and the graphs were created by Graph Pad Prism 8 (GraphPad Software, Boston, MA, USA). A significance level of 5% was considered.

## 5. Conclusions

Early administration of lipid core nanoparticles-associated methotrexate reduces NLRP1 and NLRC4 protein expression in soleus muscle without improving cardiac remodeling or changing skeletal muscle phenotype in rats.

## Figures and Tables

**Table 1:** Echocardiographic cardiac structural evaluation.

	Sham (*n* = 11)	MI (*n* = 11)	MI-MTX (*n* = 14)
LVDD (mm)	8.01 ± 0.34	11.1 ± 1.00 *	10.9 ± 1.03 *
LVSD (mm)	4.07 ± 0.61	9.34 ± 0.80 *	9.04 ± 1.28 *
IVSd (mm)	1.37 ± 0.03	1.42 ± 0.16	1.60 ± 0.29 *
AO (mm)	4.08 ± 0.11	3.89 ± 0.12 *	3.85 ± 0.12 *
LA (mm)	5.58 ± 0.26	8.15 ± 0.83 *	8.10 ± 0.90 *
LA/AO	1.37 ± 0.06	2.09 ± 0.23 *	2.11 ± 0.25 *
LVDD/BW (mm/kg)	16.74 ± 2.00	24.24 ± 1.80 *	24.48 ± 1.74 *
% of infarcted area		43.0 ± 6.21	43.0 ± 5.64
DPWT (mm)	1.37 ± 0.03	1.59 ± 0.05	1.63 ± 0.29 *

MI: myocardial infarction; MI-MTX: MI treated with methotrexate; LVDD and LVSD: left ventricular (LV) diastolic and systolic diameters, respectively; IVSd: interventricular septum diastolic thickness; AO: aorta diameter; LA: left atrium diameter; BW: body weight; DPWT: LV diastolic posterior wall thickness. Data are expressed as means ± standard deviation; ANOVA and Bonferroni or Student *t* test; * *p* < 0.05 vs. Sham.

**Table 2:** Echocardiographic left ventricular functional evaluation.

	Sham (*n* = 11)	MI (*n* = 11)	MI-MTX (*n* = 14)
Heart rate (bpm)	266 ± 38	233 ± 15	288 ± 47
FS (%)	49.3 ± 6.17	17.0 ± 3.37 *	17.9 ± 5.13 *
PWSV (mm/s)	41.0 ± 6.69	24.5 ± 6.41 *	21.4 ± 6.34 *
EF	0.88 ± 0.02	0.42 ± 0.07 *	0.44 ± 0.10 *
LV area variation (%)	72.3 ± 4.72	26.1 ± 7.60 *	26.3 ± 6.32 *
TDI S’ (average, cm/s)	3.54 ± 0.25	2.94 ± 0.36 *	2.97 ± 0.36 *
Mitral E (cm/s)	67.7 ± 11.0	77.9 ± 16.5	93.3 ± 6.82 *#
Mitral A (cm/s)	39.5 ± 9.82	27.7 ± 14.26 *	16.2 ± 1.64 *
E/A	1.76 ± 0.27	3.67 ± 2.06 *	5.80 ± 0.72 *#
EDT (ms)	46.4 (41.0–51.0)	42.0 (33.5–48.2)	36.0 (31.5–38.0) *
IVRT (ms)	26.0 (24.0–26.0)	33.0 (30.0–37.0) *	30.0 (25.0–37.0)
Tei index	0.51 ± 0.05	0.82 ± 0.14 *	0.88 ± 0.24 *

MI: myocardial infarction; MI-MTX: MI treated with methotrexate; bpm: beats per minute; FS: endocardial fractional shortening; PWSV: posterior wall shortening velocity; EF: ejection fraction; LV: left ventricle; TDI S’: tissue Doppler imaging (TDI) of systolic velocity of mitral annulus; E/A: ratio of early (E)-to-late (A) diastolic mitral inflow; EDT: E-wave deceleration time; IVRT: isovolumetric relaxation time; Tei index: myocardial performance index. Data are expressed as means ± standard deviation or medians and percentiles; ANOVA and Bonferroni or Kruskal–Wallis and Dunn; * *p* < 0.05 vs. Sham; # *p* < 0.05 vs. MI.

**Table 3:** Anatomical data.

	Sham (*n* = 11)	MI (*n* = 11)	MI-MTX (*n* = 14)
BW (g)	473 ± 48	453 ± 34	436 ± 28
LV (g)	0.88 ± 0.07	0.93 ± 0.10	0.83 ± 0.07 #
LV/BW (mg/g)	1.86 ± 0.10	2.02 ± 0.21	1.92 ± 0.11
RV (g)	0.30 ± 0.03	0.37 ± 0.08	0.48 ± 0.01 *#
RV/BW (mg/g)	0.58 ± 0.05	0.86 ± 0.20 *	1.08 ± 0.30 *#
Atria (g)	0.13 ± 0.01	0.25 ± 0.06 *	0.25 ± 0.05 *
L soleus (mg)	220 ± 30	197 ± 30	192 ± 30
R soleus (mg)	211 ± 20	192 ± 30	185 ± 30
L soleus/BW (mg/g)	0.45 (0.43–0.52)	0.41 (0.37–0.46)	0.43 (0.40–0.47)
R soleus/BW (mg/g)	0.45 ± 0.04	0.38 ± 0.14	0.42 ± 0.05

MI: myocardial infarction; MI-MTX: MI treated with methotrexate; BW: body weight; LV: left ventricle weight; RV: right ventricle weight; L: left; R: right. Data are expressed as means ± standard deviation or medians and percentiles. ANOVA and Bonferroni or Kruskal–Wallis and Dunn; * *p* < 0.05 vs. Sham; # *p* < 0.05 vs. MI.

## Data Availability

The data presented in this study are available on request from the corresponding author due to privacy and ethical restrictions.

## Supplementary Materials

The following supporting information can be downloaded at: https://www.mdpi.com/article/10.3390/ijms27094140/s1.
