# Supplementary material for: Effects of Early Treatment with Lipid Core Nanoparticles-Associated Methotrexate on Cardiac Remodeling and Soleus Muscle Inflammasomes in Infarcted Rats
Source: Int J Mol Sci. 2026 May 6;27(9):4140. doi: 10.3390/ijms27094140 (PMC13164254; doi:10.3390/ijms27094140)
Supplement: Supplementary file 1 [file ijms-27-04140-s001.zip › ijms-4229869-supplementary.pdf]

Supplementary Material

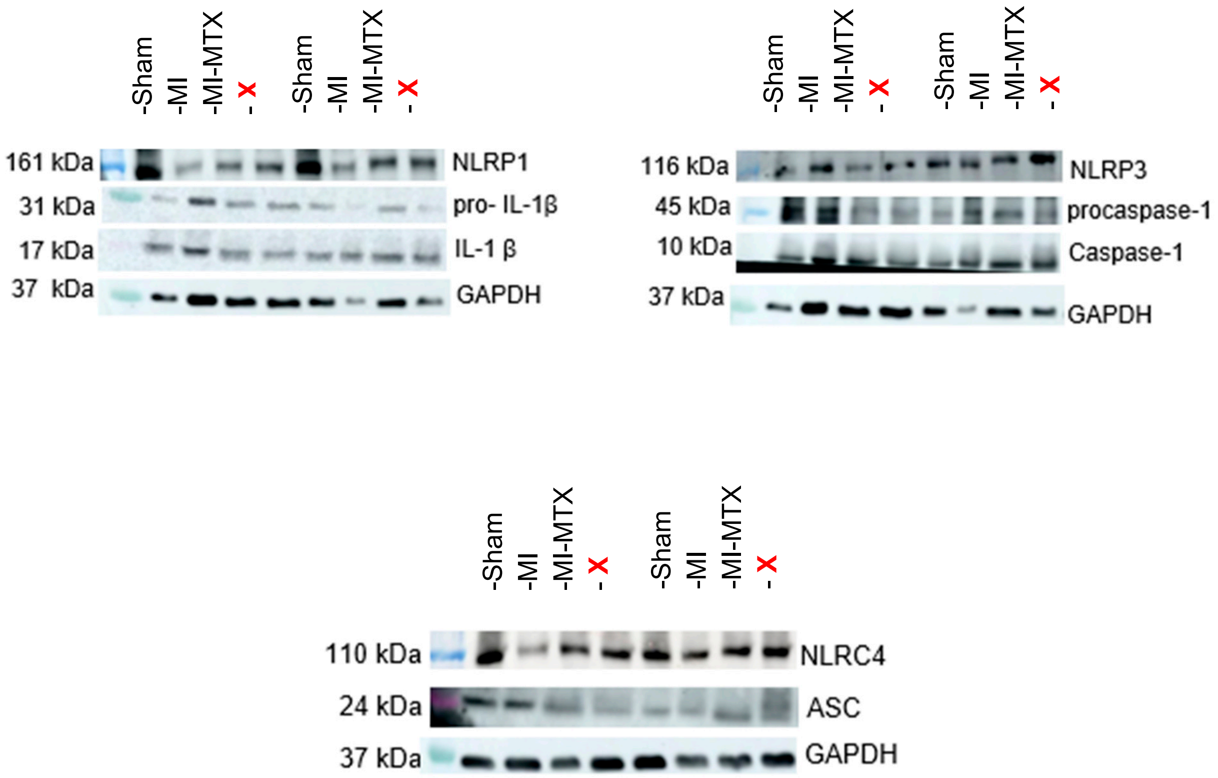

Figure S1. Representative blots of myocardial inflammasome components analyzed by Western blot. NLRP3: NACHT, LRR and PYD domains containing protein 3; Asc: apoptosis-associated speck-like protein containing a caspase recruitment domain; NLRP1: NACHT, LRR and PYD domains-containing protein 1; NLRC4: NLR family CARD domain-containing protein 4; IL-1β: interleukin-1β. MI: myocardial infarction; MI-MTX: MI treated with methotrexate; X: sample not belonging to this study.
